# Infectious Aortic Root Pseudoaneurysm after Bentall Procedure: To Treat or Not to Treat by Redo Operation?

**DOI:** 10.1055/s-0039-1694013

**Published:** 2019-10-15

**Authors:** Guglielmo Saitto, Marco Russo, Marta Pugliese, Sara R. Vacirca, Fabio Bertoldo, Paolo Nardi, Giovanni Ruvolo

**Affiliations:** 1 Department of Cardiac Surgery, Tor Vergata University Policlinic of Rome, Rome, Italy

**Keywords:** prosthetic valve endocarditis, aortic root pseudoaneurysm, redo operation

## Abstract

We present a case of a 75-year-old man who developed an early aortic bioprosthesis endocarditis due to *Klebsiella pneumoniae* complicated by aortic root pseudoaneurysm after Bentall procedure. A prompt surgical option was hypothesized, but we decided to wait and keep on clinical observation and antibiotic therapy. One year after discharge, we observed stable clinical conditions and echocardiographic findings. A question: to treat or not to treat by redo operation an infectious aortic root pseudoaneurysm?

## Introduction

Prosthetic valve endocarditis (PVE) is a complication following heart valve replacement with a reported frequency ranging from 0.3 and 1.2 per patient-year and a lifetime occurrence of 1 to 6% of patients after prosthetic valve replacement. 1

Despite advances in diagnostic options, treatment, and follow-up, the in-hospital mortality rate is still very high and more than one-third of patients die within the first year from the diagnosis. 2 3

## Case Presentation

A 75-year-old man affected by aortic root aneurysm (43 mm, with intraoperative findings of high displacement of coronary ostium, frail and thin aortic wall and an asymmetrical enlargement of the “non coronary sinus”) associated with severe bicuspid aortic valve stenosis underwent electively Bentall procedure with a composite graft constructed using a Trifecta 23 mm bioprosthesis (St. Jude Medical, MN) and a Dacron tubular 26mm prosthesis (Intervascular, Maquet, Italy). The composite conduit was implanted using multiple 2/0 nonabsorbable interrupted sutures buttressed with subannular Teflon felts passing once through the bioprosthesis annulus and Dacron conduit. Postoperative course was complicated by atrial fibrillation and cerebral ischemia with residual weakness of the lower right limb. The patient was discharged to rehabilitation in satisfactory clinical condition on the 14th postoperative day.

**Fig. 1:**
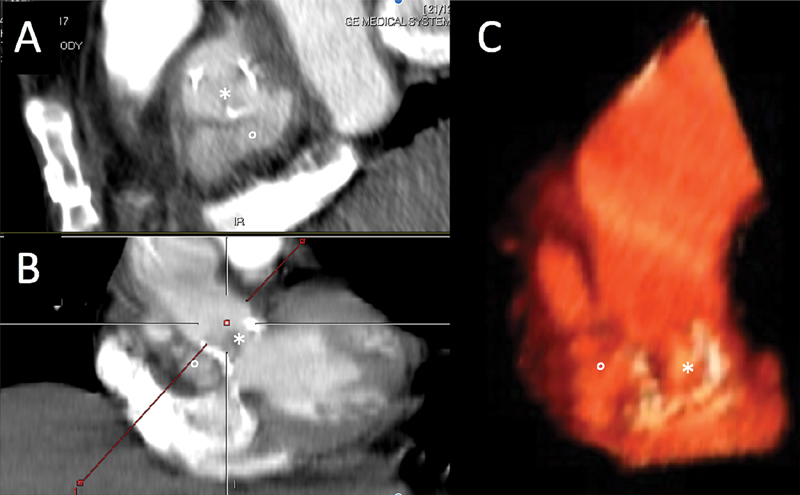
Sagittal ( **A** ) and coronal ( **B** ) plane of computed tomography scan showing the valved conduit and the pseudoaneurysm formation of the aortic root, and ( **C** ) three-dimensional volume rendering reconstruction. (*) aortic valve bioprosthesis; (°) aortic root pseudoaneurysm.

After 4 months, he was hospitalized for high fever and dyspnea. A diagnosis of PVE and pseudoaneurysm of aortic root caused by *Klebsiella pneumoniae* ESBL (extended-spectrum β-lactamase) + was made ( Fig. 1 ). The patient was transferred to our department after 1 week. Upon the arrival, he presented in a septic state, with high fever and poor general condition with altered state of consciousness, poor arousability, and confusion as to time and place. The septic state was associated with multiple organ impairment, requiring intravenous diuretic infusion and a cycle of noninvasive pulmonary ventilation. Infectious disease consultation prescribed meropenem, 2 g every 8 hours and amikacin, 1 g every 24 hours. From the beginning of the specific therapy, the blood cultures were negative. Brain magnetic resonance imaging showed embolic cerebral lesions and contrast computed tomography scan confirmed the presence of an aortic root pseudoaneurysm ( Fig. 1 ). Transesophageal echocardiography also confirmed the presence of the pseudoaneurysm but did not show a bioprosthetic valve insufficiency.

A prompt surgical consultation was obtained for the consideration of reoperation to eliminate the site of sepsis and control the perivalvular extension of the infectious disease. Considering the high surgical risk of a redo operation due to urgency setting, clinical presentation (New York Heart Association, class IV heart failure), mild renal impairment (estimated glomerular filtration rate 60 mL/min), active endocarditis, left ventricular ejection fraction 45%, pulmonary hypertension (45 mm Hg), and the complexity of the procedure (EuroSCORE II > 40%), we decided in favor of careful watching and administering prolonged antibiotic-specific therapy. After 2 weeks, a blood culture switched positive for *Candida parapsilosis* , so intravenous therapy with amphotericin B was added until negative blood culture results obtained such that the therapy with amphotericin was then stopped and switched to fluconazole. Neurological improvement, better clinical condition, and stable apyrexia were achieved in 1 month. Clinical improvement and motor rehabilitation allowed to transfer the patients after 2 months continuing intravenous antibiotic therapy (meropenem, 1 g every 8 hours and fluconazole, 200 mg every 12 hours). Repeated transthoracic echocardiograms showed a near normal bioprosthetic valve function.

After 4 weeks, the patient was discharged to home, with continuation of intravenous antibiotic therapy for a further 12-month period, with monthly blood culture. After 1-year follow-up, blood cultures were negative and the patient continued in satisfactory clinical condition. A late transesophageal echocardiography showed no paravalvular leakage and a stable dimension of the pseudoaneurysm. Even though a surgical option was considered, the patient was very frail and the operative risk remained high (EuroSCORE ≈20%); therefore, in consideration of stable clinical condition and after a long discussion, we, together with the patient and his family, decided to proceed with medical therapy.

In this case, we treated the aortic root pseudoaneurysm with prolonged antibiotic therapy and monitoring of the disease via serial blood cultures, echocardiography, and careful clinical examination. The clinical and imaging data suggest that it was possible to achieve the sterilization of the site without the need for a high-risk emergency surgery.

We will never know how this would have evolved with prompt surgical reintervention but, at least in this case, the clinical outcome thus far, with non-operative management, is quite satisfactory.

## Discussion

PVE represents a life-threatening condition, especially when complicated by perivalvular extension, like pseudoaneurysm formation of the aortic root. When this does occur, it is usually associated with severe valvular and perivalvular damage. 4 5 Other complications due to the infection are represented by ventricular septal defect, third-degree atrioventricular block, and acute coronary syndrome. 5 The best therapeutic option is still debated, but recent guidelines 6 indicate urgent surgery should be the first choice (Class of Recommendation I, Level of Evidence B) for the treatment of an uncontrolled infection, to prevent neurological or embolic complications or acute cardiac failure. However, surgical mortality remains high, up to 41% in some series. 4 5 However, for specific cases, the real-world operative risk should be considered not only by means of formal risk scores but also on the basis of the clinical status of each individual patient.

In the present case, considering the normal function of the prosthetic valve and the high risk of a redo operation in the septic state of the patient, we decided to avoid urgent surgery and to optimize medical and antibiotic specific therapies. We observed improvement in clinical condition day by day. For this reason, we decided to continue, in agreement with colleagues from infectious disease, a long-term antibiotic therapy and clinical observation avoiding redo surgery. One year after hospital discharge, the patient is still alive and in satisfactory clinical condition with negative blood cultures and stable apraxia.

This case emphasizes the critical importance of a prompt diagnosis followed by rapid medical treatment in patients with complicated PVE to avoid patient mortality and maintain hemodynamic stability. The standard approach for complicated PVE is generally represented by an urgent surgical treatment supplemented by a prolonged antibiotic treatment. In this specific case, it was possible to avoid redo urgent surgery because of the normal functioning of the bioprosthetic valve. Clinical improvement in the patient was attained by means of an aggressive medical and antibiotic specific treatment regimen, strictly monitoring the clinical state (i.e., fever) and the results of the blood cultures. At 1 year, the results seem to indicate that the right choice was made.
